# Changes in bone marrow lesions in response to weight-loss in obese knee osteoarthritis patients: a prospective cohort study

**DOI:** 10.1186/1471-2474-14-106

**Published:** 2013-03-22

**Authors:** Henrik Gudbergsen, Mikael Boesen, Robin Christensen, Else Marie Bartels, Marius Henriksen, Bente Danneskiold-Samsøe, Henning Bliddal

**Affiliations:** 1 The Parker Institute, Department of Rheumatology, Copenhagen University Hospital, Frederiksberg, Denmark; 2 Department of Radiology, Copenhagen University Hospital, Frederiksberg, Denmark; 3 Institute of Sports Science and Clinical Biomechanics, University of Southern, Odense, Denmark; 4 Faculty of Health Science, University of Copenhagen, Copenhagen, Denmark; 5 SMI, Aalborg University, Aalborg, Denmark

**Keywords:** MRI, Knee osteoarthritis, Bone marrow lesions, Weight-loss, Obesity

## Abstract

**Background:**

Patients are susceptible for knee osteoarthritis (KOA) with increasing age and obesity and KOA is expected to become a major disabling disease in the future. An important feature of KOA on magnetic resonance imaging (MRI) is changes in the subchondral bone, bone marrow lesions (BMLs), which are related to the future degeneration of the knee joint as well as prevalent clinical symptoms. The aim of this study was to investigate the changes in BMLs after a 16-week weight-loss period in obese subjects with KOA and relate changes in BMLs to the effects of weight-loss on clinical symptoms.

**Methods:**

This prospective cohort study included patients with a body mass index ≥ 30 kg/m^2^, an age ≥ 50 years and primary KOA. Patients underwent a 16 weeks supervised diet program which included formula products and dietetic counselling (ClinicalTrials.gov: NCT00655941). BMLs in tibia and femur were assessed on MRI before and after the weight-loss using the Boston-Leeds Osteoarthritis Knee Score. Response to weight-loss in BML scores was dichotomised to patients experiencing a decrease in BML scores (responders) and patients who did not (non-responders). The association of BMLs to weight-loss was assessed by logistic regressions and correlation analyses.

**Results:**

39 patients (23%) were classified as *responders* in the sum of all BML size scores whereas 130 patients (77%) deteriorated or remained stable and were categorized as *non-responders*. Logistic regression analyses revealed no association between weight-loss < or ≥ 10% and response in BMLs in the most affected compartment (OR 1.86 [CI 0.66 to 5.26, p=0.24]). There was no association between weight-loss and response in maximum BML score (OR 1.13 [CI 0.39 to 3.28, p=0.81]). The relationship between changes in BMLs and clinical symptoms revealed that an equal proportion of patients classified as BML *responders* and *non-responders* experienced an OMERACT-OARSI response (69 vs. 71%, p=0.86).

**Conclusions:**

Weight-loss did not improve the sum of tibiofemoral BML size scores or the maximum tibiofemoral BML score, suggesting that BMLs do not respond to a rapidly decreased body weight. The missing relationship between clinical symptoms and BMLs calls for further investigation.

## Background

The incidence of obesity has steadily increased over the past decades and combined with the fact that patients are susceptible for knee osteoarthritis (KOA) with increasing age and obesity KOA is expected to become a major disabling disease in the future [1-3]. It is highly relevant to examine the effect of weight-loss in obese KOA patients as this modifiable factor could have an effect on KOA-related structural changes, as assessed by magnetic resonance imaging (MRI).

Clinical symptoms of KOA are increased in obese individuals and it is known that weight-loss can both improve symptoms as well as decrease the risk of KOA development [4-7]. A weight reduction of 10% and above has been shown to significantly improve the KOA-related symptoms regardless of the severity of structural KOA changes as assessed by MRI [8-10].

At present, KOA is considered to be a whole organ disease that affects cartilage, bone marrow, ligaments, menisci, joint capsule etc. and this can be assessed by MRI scans using the BLOKS (Boston-Leeds Osteoarthritis Knee Score) [11]. In general, an inconsistency exists between symptoms and imaging but specific MRI assessed pathology has been shown to correlate to clinical symptoms and/or progression of disease [12-17]. A very important feature is changes in the subchondral bone which may be evaluated by BLOKS as bone marrow lesions (BMLs). BMLs have been associated to the future degeneration of the joint in KOA, prevalent clinical symptoms and malalignment by means of an increased mechanical load [13,18-21].

The aim of this study was to investigate the changes in BMLs after a 16-week weight-loss intervention in obese subjects with KOA and relate changes in BMLs to the symptomatic effect of weight-loss. We hypothesized that weight-loss would improve the overall and maximum BML scores on MRI and that the effects of weight-loss on BMLs would be related to changes in clinical symptoms.

## Methods

### Patient population

192 participants were recruited November 2007–August 2008 from the outpatient clinic at the Department of Rheumatology, Frederiksberg Hospital, Denmark, to take part in the CAROT study (ClinicalTrials.gov identifier: NCT00655941) [22]. Screening of possible participants was performed by a formalized telephone interview. Of the 388 screened subjects 187 patients were ineligible and 9 patients declined to participate in the trial. 192 patients remained for inclusion to this prospective cohort study; further details have been published [22].

To be eligible for inclusion, individuals had to be over 50 years of age, have a body mass index (BMI) ≥ 30 kg/m^2^, and show primary KOA diagnosed according to the American College of Rheumatology (ACR) criteria combining clinical symptoms as well as radiographic verification of the diagnosis [23].

All patients signed and approved an informed consent. At baseline and follow-up (week 16) we performed conventional 1.5T MRI, whereas radiographs were only obtained at baseline.

The study was approved by the local ethical committee of The Capital Region of Denmark [H-B-2007-088] and was carried out in accordance with the Helsinki Declaration II and the European Guidelines for Good Clinical Practise.

### Interventions

The patients followed a supervised 16-week dietary program consisting of nutritional education and a diet of normal food plus meal replacements (The Cambridge Diet, the Cambridge Health and Weight plan UK). The details of the dietary program is described elsewhere [22]. The dietary program consisted of an 8-weeks intensive weight-loss phase with an all provided formula diet. In the initial 8 weeks the patients were randomized to either Low Energy Diet (LED) or Very Low Energy Diet (VLED), corresponding to 810 kcal/day or 485 kcal/day. This was followed by an 8-week period with part formula/part conventional food.

### MRI acquisition

MRI scans were obtained of the target knee (worst symptomatic knee) using a high-field MRI (1.5 T) whole body scanner (Philips Intera; software release 12.1.5.0) following normal procedure taking one scan at each setting. Patients were positioned supine and a send/receive flex medium or large coil was fixed to the patient’s leg. The study crew ensured that patients were scanned using the same image and coil setup over time. The following five sequences were carried out:

Gradient echo-scout (10 mm slices, repetition time (TR) 12.3 ms, echo time (TE) 6.6 ms, 50° flip angle, field of view (FOV) 300 x 300 mm, matrix 256 x 256). Sagittal 3D T1-FFE gradient-echo (GRE) cartilage sequence (3 mm slices, TR 21 ms, TE 8.4 ms, 20° flip angle, FOV 160 x 160 mm, matrix 512 x 512). Sagittal multi-echo T2 weighted sequence (4 mm slices, TR 2531.3 ms, TE 100 ms, FOV 170 x 170 mm, matrix 256 x 256). Sagittal multi-echo proton density (PD) weighted sequence (4 mm slices, TR 2531.3 ms, TE 15.4 ms, FOV 170 x 170 mm, matrix 256 x 256). Coronal T1 spin echo (SE) sequence (3 mm slices, TR 500 ms, TE 17 ms, FOV 150 x 150 mm, matrix 512 x 512 ). Coronal STIR (short tau inversion recovery) sequence 3 mm slices (TR 1797.9 ms, TE 55 ms, FOV 150 x 150 mm, matrix 512 x 512). Scheduled time for the overall MRI examination was 37 minutes.

### MRI assessments

The imaging protocol used for the BLOKS assessment in this study was the sagittal multi echo PD and T2 weighted scans, the STIR scan, the coronal SE T1 scan, as well as a 3D reconstruction of the T1-FFE scan.

BMLs appear as ill-defined signal intensity changes in the subchondral bone that are hypointense on T1w images and hyperintense on STIR images [24]. BML assessments were done semi-quantitatively in 7 of the 9 regions of the knee, excluding the patellar regions. Analyses in this study included data from 6 regions as we discarded the BML assessments in the tibial intercondylar region. BMLs were graded on a 0–3 scale based on the extent of regional involvement; 0=none, 1=less than 10% of the region; 2=10% to 25% of the region; 3= more than 25% of the region. Cartilage assessments were performed using the sagittal GRE sequence which was transformed to a 3D multi planar reconstructed (MPR) scan with near isotropic voxels [25,26]. Osteophytes were evaluated using all three planes. In the axial plane we scored lateral and medial osteophytes on patella as well as anterior and posterior osteophytes on femur using the axial reconstructed 3D gradient echo sequence. In the coronal plane we assessed central weight-bearing osteophytes on tibia and femur. In the sagittal plane we examined the anterior and posterior osteophytes on femur and tibia, as well as the superior and inferior osteophytes on patella [11]. For the evaluation of menisci we analysed morphology, tears and extrusion on the coronal T1w TSE (body) and on the sagittal T2w/PDw sequences (anterior and posterior horns).

Initial inter- and intra-reader analyses were performed by HG and MB by scoring 20 consecutively selected scans from the 187 completed baseline MRI examinations. Selection was performed so that the analyses were completed on 10 females and 10 males. The selected patients displayed all levels of KOA joint damage, evaluated by the medial compartment KL grade on radiographs (2 patients having KL grade 0, 6 patients having KL 1, 4 patients having KL 2, 6 patients having KL 3, and 2 patients having KL 4). This procedure was similar to what was done in the original BLOKS publication; please see the online appendix [11]. Discrepancies in the initial training period were resolved at meetings held by MB and HG. Following this HG performed all the BLOKS assessments.

All imaging assessments were performed using the MacOS X based Osirix software (version 3.9.1) [27].

### Radiographic assessments

Bi-plane weight-bearing semi-flexed radiographs were taken of the index knee; one in the postero-anterior and one in the lateral view (in case of bilateral symptoms we used the most symptomatic knee). The radiographs were obtained at inclusion by the same radiographers, using a Philips Optimus apparatus and a standardized protocol through all examinations.

MB analysed all radiographs using an atlas [28] and performed a separate score for each knee compartment according to the KL grading system [29]. As the original grading by KL did not deal with the assessment of the patellofemoral compartment we aimed to perform and explore a whole joint radiographic assessment and thus applied the KL criteria for tibiofemoral KOA to the patellofemoral compartment [30]. The reported KL, for the knee in general, was calculated as the maximum score in either chamber.

Measurements of the tibiofemoral minimum joint space width (mJSW) in the most affected tibiofemoral compartment, judged by the KL grade, were performed by HG. Inter- and intra-reader analyses of the mJSW were performed by scoring the same scans as described above for the inter- and intra-reader analyses of BLOKS [10].

### Knee joint alignment axis

Mechanical axis alignment was measured using a 6 camera stereophotogrammetric system (Vicon MX, Vicon, UK) with markers placed on anatomical landmarks (2^nd^ metatarsal head, lateral malleolus, posterior aspect of calcaneus, lateral aspect of the leg, lateral femoral epicondyle, lateral aspect of the thigh, bilaterally on anterior and posterior superior iliac spines) according to the Plug-in-Gait biomechanical model, and anthropometric measurements (height, leg length, and knee and ankle diameters) to determine joint centres. The mechanical axis alignment was defined as the frontal plane knee joint angle expressed in the local joint coordinate system. This procedure yields estimates of mechanical axis alignment similar to full-limb weight-bearing radiographs (R^2^=0.54) but without exposure to radiation [31]. A knee was defined as a varus when alignment was > 0º and valgus when < 0º.

### Isometric maximal voluntary contraction

Isometric maximal voluntary contraction (MVC) of hamstrings and the quadriceps muscles were assessed by isometric dynamometry at 60° (0° is full extension) knee joint flexion angle (Biodex System 3 PRO, Biodex Medical System, NY, USA) as described [32]. After calibrating the system, the subject was comfortably seated and fastened to the dynamometer chair with leg- and body-straps. Prior to the measurements, a correction for gravity was made by registering the leg’s weight at 0° knee joint angle. The protocol was comprised of 6 successive maximal efforts alternating between knee extension and knee flexion. Each contraction lasted 5 seconds with a 10 second pause between contractions. After test trials, performed to familiarize the patients to the test, the average peak value of three trials was chosen as MVC. Vigorous verbal encouragement was given in an attempt to achieve maximal effort level. Isometric MVC-values were normalized to body mass (Nm/kg).

### Symptomatic assessments

Patient reported outcomes were examined at baseline and after 16 weeks by assessing the OMERACT-OARSI Responder Criteria and KOOS pain and function in daily living (ADL), which were completed by the patients on a validated touch screen solution [33].

The OMERACT-OARSI responder criterion was assessed by average pain, disability and patient global assessment of disease impact during the last week using 0 to 100 mm visual analogue scales (VAS) [34]. The Knee Injury and Osteoarthritis Outcome Score (KOOS) was used to assess impairment, disability and handicap in 5 sub domains (function in daily living (ADL), pain, knee-related quality of life, symptoms, and function in sport/recreation). Items are scored from 0–4 and then transformed into a 0–100 scale; 0 representing extreme knee-related problems and 100 representing no knee-related problems [35].

### Statistical methods

Analyses were performed on available cases (*per protocol analysis*). The most affected knee compartment was the one with the worst status on radiographs and only this single compartment for every patient case was included in the analyses exploring the relationship between weight-loss and BML responses. In order to examine the importance of changes observed in BML gradings we calculated the least significant criterion (LSC) using the following formula LSC= 1.96 * SE * √ 2 * (1-r) (r; serial correlation) [36]. To overcome that there can be several BMLs in each region the BML scores were summed and this overall score was considered for analyses together with the maximum BML score.

We dichotomised the observed changes and our dependent variable contained 0 for patients who progressed or remained stable (non-responders) in their BML scores and 1 for patients responding positively (responders) [37]. As a sensitivity analysis we performed ancillary statistical testing only including patients with a BML score > 0 at baseline. Changes in body weight was analysed as relative measures, being the percentage change from baseline ((x_16_ - x_0_)/x_0_ * 100%). Regarding the influence of weight-loss on BMLs we conducted both regression and correlation analyses. A logistic regression model analysed the influence of a weight-loss < or ≥ 10% of the initial body weight on the response in BML scores. These regressions were repeated with adjustments for age, gender and group allocation in the underlying randomisation to either the LED or VLED intervention. The correlation analyses examined the relationship between weight-loss, as a continuous variable, and the response in BML scores. Changes in symptomatic outcomes (KOOS and OMERACT-OARI response) were analysed as relative measures, being the percentage change from baseline ((x_16_ - x_0_)/x_0_ * 100%), and were then analysed in relation to the dichotomised response in BML scores.

The inter- and intra-reader analyses of the mJSW were performed by applying an ICC two-way mixed model with measures of absolute agreement whereas the inter- and intra-reader analyses of BLOKS were performed by weighted kappa analyses based on individual scores.

A P-value less than 0.05 (two-tailed) or a 95% confidence interval (CI) not including the null hypothesis was regarded as statistically significant. All data analyses were carried out applying SAS software (v. 9.2; SAS Institute Inc., Cary, NC, USA).

## Results

The study included 192 obese KOA patients and following the 16 weeks of diet intervention 175 (%) patients remained in the study. 187 (97%) MRI scans were completed at baseline, 172 (98%) MRI scans were obtained at week 16 and this left the study with 169 (97%) patients with complete MRI datasets. No statistical significant differences were detected between baseline characteristics of all the initially included patients (n=192) and the 169 patients included in the analyses in this paper (p<0.05).

The majority of patients were female (80%) and an average participant had a BMI of 37.0 kg/m^2^ (SD 4.5) and was 62.7 years (SD 6.3) of age (Table 1). One in six patients did not have any BMLs at baseline. Radiographic assessments showed that the majority of patients had a diminished joint space, compared to normal patients, and 81% were classified as having KL grade 1–3. Judged by radiographs 53 patients had unicompartmental medial tibiofemoral KOA (defined as a medial KL ≥ 2 and a lateral KL ≤ 1) whereas only 12 patients had solely lateral tibiofemoral KOA.

**Table 1:** Baseline characteristics of all participants

**Basic characteristics**	**N = 169**
Female no. (%) ^*^	136 (80 %)
Age (years) ^*^	62.7 ± 6.3 (49.6-76.5)
Height (cm) ^*^	166 ± 8 (148–191)
Weight (kg) ^*^	102.3 ± 14.6 (76.0-145.0)
BMI (kg/m^2^) ^*^	37.0 ± 4.5 (30.1-51.6)
C-reactive protein (mg/L) ^1 §^	4.1 [2.2;7.3] (0.7-58.6)
**Isometric maximal voluntary contraction**	
Quadriceps Muscle Strength (nm/kg) ^1, 2 +^	1.1 [0.9;1.4] (0.1-2.8)
Hamstrings Muscle Strength (nm/kg) ^1, 2 +^	0.5 [0.4;0.6] (0.0-1.1)
**Radiographs**	
KL _whole joint score_ (0–4) ^1, 3 *^	3.0 [2.0;3.0] (1.0 – 4.0)
KL _medial tibiofemoral joint_ (0–4) ^1, 3 *^	2.0 [2.0;3.0] (0.0 – 4.0)
KL _lateral tibiofemoral joint_ (0–4) ^1, 3 *^	2.0 [1.0;2.0] (0.0 – 4.0)
KL _patellofemoral joint_ (0–4) ^1, 3 *^	2.0 [1.0;3.0] (0.0 – 4.0)
mJSW ^1, 4 ^^	2.2 [0.00;3.8] (0.0 – 7.3)
**MRI**	
Bone Marrow Lesions _sum of size scores_^1, 5 *^	3.0 [1.0;5.0] (0.0-9.0)
Cartilage I _combo score_(0–81) ^1, 6 *^	15.0 [6.0;27.0] (0.0-61.0)
Osteophytes _sum of scores_ (0–36) ^1 *^	13.0 [8.0;18.0] (1.0-35.0)
Menisci _sum of extrusion scores_ (0–12) ^1 *^	7.0 [5.0;9.0] (0.0-12.0)
Menisci _sum of morphology scores_ (0–38) ^1 *^	7.0 [6.0;8.0] (0.0-11.0)
**Symptomatic assessment**	
KOOS _Function in daily living (ADL)_^*^	60.4 ± 16.6 (11.8 – 98.5)
KOOS _Pain_^*^	57.4 ± 15.9 (11.1 – 100)
**Knee joint alignment axis **^**7**^	
Varus (>0) / Valgus (<0) º	6.0 ± 4.8 (-8.6 – 23.7)

Plus-minus values are means ± SD and (min-max) unless otherwise stated.

^1 ^Presented as median, interquartile range [Q_1_;Q_3_] and (min – max).

^2 ^Measured isometric at 60 ° and normalized to body weight (nm/kg).

^3 ^Scored using the Kellgren & Lawrence Grading system.

^4 ^mJSW; minimum Joint Space Width (mm).

^5 ^Sum of all BML size scores except those from patella.

^6 ^For all analyzed areas we multiplied the “surface area”-score (0–3) with the “full thickness”-score (0–3), reaching results between 0 and 9 for each area, and then multiplied them into one “combo score” reaching a final score between 0 and 81.

^7 ^From the static anatomical landmark calibration trials for each subject the knee joint mechanical axis alignment was calculated using a Plug-In-Gait model.

Number of participants included in analysis varied due to missing data for specific variables, as follows: ^* ^169 patients; ^+ ^164 patients; ^^^ 152 patients; º 158 patients; ^§ ^162 patients.

The inter- and intra-reader analyses of the subscores in BLOKS revealed kappa values between 0.51-0.90 (Table 2) and the inter- and intra-reader analyses of mJSW showed ICCs between 0.93 and 0.98, respectively. The LSC for the total BML size score was < 0.5 and any change in BML gradings was therefore considered to be significant.

**Table 2:** Weighted kappa and CI for inter- and intra-reader reliability

	**Inter-reader**^**1**^	**Intra-reader**^**1**^
**Cartilage I**	0.59 (0.31;0.87)	0.81 (0.71;0.91)
**Cartilage II**	0.71 (0.42;1.00)	0.75 (0.62;0.88)
**Bone Marrow Lesions**	-	-
Size score	0.65 (0.47;0.83)	0.66 (0.56;0.76)
Percentage BML score	0.72 (0.50;0.94)	0.76 (0.64;0.88)
Adjacency score	0.70 (0.48;0.92)	0.78 (0.66;0.90)
**Osteophytes**	0.71 (0.48;0.94)	0.81 (0.82;0.90)
**Effusion**	0.51 (0.21;0.81)	0.72 (0.61;0.83)
**Synovitis **(whole knee)	0.66 (0.42;0.90)	0.70 (0.60;0.80)
**Synovitis **(subscale)	0.61 (0.27;0.95)	0.58 (0.41;0.75)
**Meniscal Extrusion**	0.63 (0.38;0.88)	0.67 (0.55;0.79)
**Meniscal Signal**	0.80 (0.54;1.00)	0.80 (0.67;0.93)
**Meniscal Tear**	0.70 (0.38;1.00)	0.90 (0.80;1.00)

^1 ^Validated on 20 patients consecutively selected according to protocol.

39 patients (23%) experienced a decrease in the sum of all BML size scores (*responders*) and 130 patients (77%) deteriorated in their score or remained stable (*non-responders*) (Table 3).

**Table 3:** Changes in BMLs and clinical symptoms as a response to weight loss

	**Per protocol analyses**		**Per protocol analyses**	
	**Patients with baseline BML score ≥ 0**		**Patients with baseline BML score > 0**	
	**N = 169**		**N = 139**	
	**Weight loss**	**Weight loss**	**Weight loss**	**Weight loss**
	**< 10 %**	**≥ 10 %**	**< 10 %**	**≥ 10 %**
**Change in the *sum of all BML scores***				
Responders	N = 5 *(15 %)*	N = 34 *(25 %)*	N = 5 (*20 %)*	N = 34 (*30 %)*
Non-responders	N = 29 *(85 %)*	N = 101 *(75 %)*	N = 20 *(80 %)*	N = 80 *(70 %)*
**Change in the *maximum BML score***				
Responders	N = 5 *(15 %)*	N = 23 *(17 %)*	N = 5 *(20 %)*	N = 23 *(20 %)*
Non-responders	N = 29 *(85 %)*	N = 112 *(83 %)*	N = 20 *(80 %)*	N = 91 *(80 %)*
**OMERACT-OARSI response**				
Responders	N = 16 *(47 %)*	N = 103 *(76 %)*	N = 13 *(52 %)*	N = 87 *(76 %)*
Non-responders	N = 18 *(53 %)*	N = 32 *(24 %)*	N = 12 *(48 %)*	N = 27 *(32 %)*

**Figure 1:**
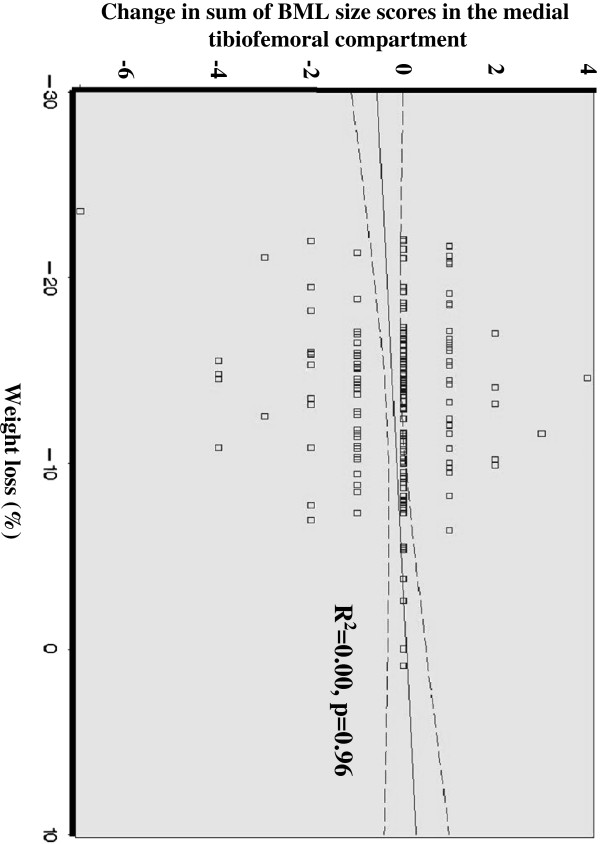
The relationship between weight-loss and changes in medial tibiofemoral BML size scores.

Logistic regression analyses revealed no association between weight-loss category and response in BML size in the most affected compartment (OR =1.95 [CI 0.70 to 5.45, p=0.20]). Adjusting for age, gender and randomization group did not change the results significantly (OR 1.86 [CI 0.66 to 5.26, p=0.24]). There were no association between weight-loss during the diet intervention period and the response in maximum BML score (OR 1.13, CI 0.39 to 3.28, p=0.81). Focusing the analyses to only include patients having a baseline BML score > 0 (N = 139) did not have any important impact on the results (Table 3). Analysing the association between percentage weight-loss, as a continuous variable, and changes in BMLs in the worst knee compartment did not show any relationship between the two measures (Figure 1).

There were no differences in BML responses in the underlying RCT (data not shown).

The relationship between changes in BMLs and clinical symptoms revealed that an equal percentage of patients classified as BML *responders* and *non-responders* experienced an OMERACT-OARSI response (69 vs. 71%, p=0.86). KOOS pain improved 23.9 and 22.6% for BML *responders* and *non-responders*, respectively (mean difference 1.3 [95% CI -14.3 to 11.7], p=0.84) and similar results were found for KOOS ADL (25.6 and 25.7%, mean difference 0.1 [95% CI -14.2 to 14.3], p=0.99). Comparable results were found when analysing the association between response in maximum BMLs and KOOS pain (16.3 and 24.2%, mean difference -7.9 [95% CI -6.8 to 22.6], p=0.29) as well as KOOS ADL (20.5 and 26.7%, mean difference -6.2 [95% CI -10.0 to 22.3], p=0.45).

## Discussion

In this study we aimed to assess whether or not a 16-week diet intervention program would impact on BMLs, and we assessed both the maximum and overall BML scores. Results from the study were that changes seen in the total tibiofemoral sum of BML scores and maximum BML scores were not impacted by weight loss. Also, we found no relationship between improvements in clinical symptoms and BMLs following a weight-loss.

BMLs are a common finding in painful osseous conditions and have been proposed to consist of extracellular fluid that causes pain via an increased intraosseous pressure [38,39]. In cross sectional studies BMLs seem to be the MRI feature most consistently associated to clinical KOA symptoms [13,40,41], while only one longitudinal study have shown this [20,42]. Furthermore, it has repeatedly been shown that BMLs predicts cartilage loss and overall progression of disease [18,19].

Prior to the intervention our cohort had a high prevalence of BMLs, which is somewhat in contrast to previous reports investigating symptomatic KOA patients [37,43]. In contrast to these trials Felson et al. revealed results which are similar to those reported from this trial [44]. Previous data have reported that BMLs fluctuate over time. The development in total sum score of BML sizes in the present study was comparable to results from the MOST study and other studies, while BMLs in general developed more positive in our study population when compared to data from other prospective observational cohorts [37,42,43,45]. The differences might be due to the very rapid and successful weight-loss used in our study, which was applicable with high intensity with few adverse effects [22].

The present study did not provide any biological explanation for the response in BMLs after weight-loss. Data from the MOST study suggest an association between alignment of the knee and progression of BML mediated through increased mechanical load in a compartment-specific manner, since alignment is believed to influence load distribution [21]. The present results contrast this, because the changes in BMLs were not associated to the mechanical unloading (weight loss). Although the present study was not focused on alignment, it indicates that the suggested association between alignment and BML progression [21] is not mediated through mechanical loadings (Table 3). Existing literature suggests that obesity related inflammatory mechanisms and biomechanical malfunction are both related to structural damage in the knee, but even so, the aetiopathogenesis of BMLs is still not fully understood [46-48]. Lacking the complete overview of mechanisms related to KOA might explain the absent capacity of this study to show an effect of weight loss on BMLs.

Neither did the study provide data clarifying the relationship between BML changes and the effects of weight-loss on clinical symptoms. Several factors are likely to have an impact on the assessments and interpretations of clinical symptoms in KOA. First of all, longitudinal changes in BMLs have not been shown to correlate with corresponding changes in KOA symptoms [42]. Secondly, as patients are subjected to KOA symptoms over a long period of time, the patients may develop peripheral joint sensitization as well as plasticity changes in the central nervous system [49,50] that could disturb the relationship between structural damage and symptoms [46]. Thirdly, the overall levels of cytokines and adipokines have an impact on clinical symptoms. Last but not least, the subjective experience of pain is influenced by cognitive, social, emotional and behavioural factors; e.g. expectations, general anxiety, and previous experience [51,52]. All of these considerations are likely to influence the clinical observations and could explain the lack of association between changes in symptoms and MRI-graded pathology.

The inter- and intra-reader analyses of both the BLOKS assessments and the mJSW showed results that were comparable to those achieved by others [11,53,54].

The study has some limitations. The agreement on when an observed change is important is not yet clarified for BMLs assessed by BLOKS, but we believe that our estimate of the LSC validated our results. Our analyses are based on single determinations of MRI items which may result in erroneous estimations of associations due to the subjective variability of these. The MRI protocol for this study did not include all the recommended sequences for optimal gradings of KOA pathology. However, we believe that the BLOKS assessment performed in general served the question addressed in this study. The protocol only included coronal slices for the assessment of BMLs in trochlea and the dorsal portion of the femoral condyles and even though we consider this to be reasonable [55,56] it is likely not to be optimal. In general, we consider the coronal STIR and T1w sequences adequate for a reasonable assessment of BMLs in the tibial and femoral bones as Osirix allowed for a localization of the scored lesions by using sagittal sequences obtained for other purposes. However, we recognize the limitations this strategy withholds in terms of correctly assessing BMLs located at the margins of our slices. Due to unacceptable coverage of patella, data did not include BML scores from this part of the knee joint. Also, we chose to sum the scores of individual assessments of cartilage pathology, BMLs and menisci to form a sum-score for each of the three compartments and to exclude scores from the tibial intercondylar region [57-59]. With respect to the KL gradings a limitation is that we did not perform intra observer variation of the KL grading. Finally, changes assessed over period of just 16 weeks may be too short a time frame for assessing changes in BMLs, even though preliminary data support that this may in fact occur [44].

## Conclusions

Weight-loss did not improve the total sum of tibiofemoral BML size scores or the maximum BML score, suggesting that BMLs do not respond to a rapidly decreased body weight. Ancillary sensitivity testing that excluded patients with no BMLs at baseline showed similar results and thus supported the initial findings.

In conclusion, this study did not show any association between weight-loss and response in BML scores and failed to connect a positive BML response to clinical improvements. The missing relationship between changes in clinical symptoms and BMLs calls for further investigation.

## Abbreviations

ACR: American college of rheumatology; BLOKS: Boston-Leeds Osteoarthritis Knee Score; BMI: Body mass index; BML: Bone marrow lesion; CAROT: Influence of weight-loss or exercise on cartilage in obese knee osteoarthritis patients: a randomized controlled trial; KOA: Knee osteoarthritis; FOV: Field of view; GRE: Gradient echo; ICC: Intraclass correlation coefficient; KL: Kellgren & Lawrence grade; KOOS: Knee Injury and Osteoarthritis Outcome Score; LED: Low energy diet; LSC: Least significant criterion; mJSW: Minimum joint space width; MPR: Multi planar reconstructed; MRI: Magnetic resonance imaging; MVC: Maximal voluntary contraction; OR: Odds-ratio; PD: Proton density; SE: Spin echo; STIR: Short tau inversion recovery; TE: Echo time; TR: Repetition time; VAS: Visual analogue scale; VLED: Very low energy diet.

## Competing interests

HB, RC and MH received travel grants to attend scientific meetings from the Cambridge Manufacturing Ltd. The sponsor had no involvement with respect to design, collection of data, analysis, interpretation, writing or submission.

## Authors’ contributions

HG made all the analysis and interpretation of data, drafted the manuscript and approved the final version. MB contributed to the conception and design, analysed all MRI and radiographs, revised the manuscript several times and approved the final version. RC contributed to the conception and design, especially the statistics, revised the manuscript and approved the final version. EMB, MH and BDS contributed with the overall design idea, revised the manuscript and approved the final version. HB contributed to the conception and design, revised the manuscript and approved the final version.

## Pre-publication history

The pre-publication history for this paper can be accessed here:

http://www.biomedcentral.com/1471-2474/14/106/prepub
